# Behavioural and physiological responses to stressors in sheep with temperament classified by genotype or phenotype

**DOI:** 10.1038/s41598-024-58959-y

**Published:** 2024-04-08

**Authors:** Yuri Kitagawa, Shane K. Maloney, Kelsey R. Pool, Dane Webster, Satoshi Ohkura, Dominique Blache, Luoyang Ding

**Affiliations:** 1 College of Animal Science and Technology, Yangzhou University, Yangzhou, 225009 China; 2 Laboratory of Animal Production Science, Graduate School of Bioagricultural Sciences, Nagoya University, Togo-cho, Aichi, Japan; 3 School of Agriculture and Environment, M087, The University of Western Australia, 35 Stirling Highway, Perth, WA 6009 Australia; 4 School of Human Sciences, M309, The University of Western Australia, 35 Stirling Highway, Perth, WA 6009 Australia

**Keywords:** Physiology, Biomarkers, Endocrinology

## Abstract

The single nucleotide polymorphism (SNP) rs107856856, located in the tryptophan hydroxylase-2 gene, is associated with the behavioural phenotype for sheep temperament measured at weaning. Here, we tested the association between that SNP and physiological and behavioural responses to stressors in adult sheep. Two groups of adult sheep, one with genotype A/A (calm genotype) and the other with G/G (nervous genotype) in rs107856856, were selected from 160 sheep and were exposed, twice, to an open-field arena and an isolation box test (IBT). During each repeat, the behaviour and physiological responses (cortisol, prolactin, dehydroepiandrosterone [DHEA], brain derived neurotrophic factor [BDNF], characteristics of the response of body temperature, and oxidative stress) were measured. The behavioural and physiological responses of the sheep were compared between genotypes and also between groups classified on their phenotype as assessed by their initial isolation box score (“low responders” and “high responders”). The SNP rs107856856 had some effects on the behavioural phenotype (IBT score) but no effects on the physiological response to stress (cortisol, prolactin, DHEA, BDNF, oxidative stress or changes in body temperature) in the adult sheep, probably because the sheep were exposed, and therefore had adapted, to human contact during their life.

## Introduction

Animal temperament is a complex trait that can be defined as the consistent behavioural and physiological responses that are expressed by an animal when it is exposed to an eliciting event. Temperament exists on a continuum that has been described in many ways, classifying animals as more-reactive v less-reactive, temperamental v placid, difficult v easy, excitable v docile, reactive v quiet^1^. In this paper, we will use the descriptors “nervous” and “calm” to define temperament in sheep. The genetics that underlie temperament has been investigated using association studies between genetic variants and the behavioural responses of individuals to a stressor. In cattle, many single nucleotide polymorphisms (SNPs) and candidate genes have been associated with temperament where temperament has been categorised by the behavioural responses of the animals to the approach of a human, contact with a human, handling by a human, or social isolation^2–7^. Similarly, in young Merino sheep, several genetic variants have been associated with the behavioural response of the animals to human contact and/or social isolation around weaning, including SNPs located in genes that encode for dopamine receptor 2, steroid 17α-hydroxylase/17,20-lyase, the serotonin receptor, and tryptophan hydroxylase-2 (TPH2)^8–10^. Nearly all these studies on the genetic basis of temperament have focused on the association between SNPs and behaviour. To date there has been no study on the genetics that might be associated with physiological responses to a stressor. It is likely that variation in the physiological responses of animals to a stressor will be more important to production performance than will variation in behavioural responses^11^. That would not be an issue if the behavioural and physiological responses were perfectly correlated, but there is no evidence that they are always positively correlated, and even some evidence that the behavioural and physiological responses to stress are negatively correlated. For example, individual sheep that had a relatively higher response of the hypothalamic–pituitary–adrenal axis to a stressor, which would categorise those animals as more nervous, tended to display withdrawal behaviour, which would categorise those animals as calm^12,13^. Therefore, it is necessary that any search to uncover the genetics of temperament should incorporate the physiological response to stress, together with the behavioural response, so that potential impacts on productivity can be assessed.

Of the potential genetic variants in sheep that might underlie variation in temperament and the physiological responses to a stressor, the neurotransmitter serotonin emerges as a prime candidate. Serotonin plays an important role in the brain pathways that activate the main physiological responses to a stressor. Supplementation of serotonin in fish and mice inhibits the release of stress hormones, including cortisol and prolactin^14–16^. In dogs, the concentration of serotonin in the blood is negatively correlated with the concentration of dehydroepiandrosterone (DHEA) which is another important stress hormone^17^. Moreover, serotonin interacts with brain-derived neurotrophic factor (BDNF), which is a significant factor that affects brain ontogeny and the response to stressors, in the expression of several behavioural disorders in humans^18^. Together, these results suggest that serotonin and its signalling pathway acts as a “calming” drive in mammals.

Variation in the gene that encodes for the enzyme that limits the rate of production of serotonin, the TPH2 gene, can affect the release of stress hormones^19–21^. Variation at SNP A2051C in TPH2 in rhesus monkeys led to variation in the expression of the TPH2 gene and differences in the activity of the hypothalamic–pituitary–adrenal (HPA) axis. Individual monkeys that were homozygous at SNP A2051C (C/C) had lower expression of TPH2 and a higher baseline cortisol and a higher response to adrenocorticotropic hormone, than did the individuals that were homozygous A/A or heterozygous A/C^22,23^. Interestingly, serotonin has been implicated in the oxidative stress that is associated with depression in humans and depression like syndrome in rodents^24^. For example, the administration of a precursor of serotonin, or blockage of the re-uptake of serotonin, decreased both oxidative stress and depression like syndrome in humans^25^.

The serotonin pathway has also been implicated in the regulation of core body temperature (T_c_) and stress-induced hyperthermia (SIH) that has been shown to be a thermal indicator of psychological stress^26,27^. Mice with genetic ablation of the serotonin receptor have a smaller decrease in T_c_ when they were injected with selective agonists of the serotonin receptor^28^. Similarly, genetically modified mice with almost complete ablation of serotonin neurons had higher T_c_ than did the wildtypes, and the knockouts were also more active^29^. Knock out of the serotonin transporter (SERT) attenuates the SIH response, while blockade of the 5-HT1A receptor increases the SIH response^30^.

The data presented above strongly suggest that SNP rs107856856 in the TPH2 gene in Merino sheep could affect the physiological response to a stressor, including changes in body temperature, cortisol, prolactin, DHEA, and BDNF, in addition to the behavioural responses that have already been studied^10^. In the present study, we aimed to test whether there were differences in the physiological responses to stress between sheep that were classified as calm or nervous in two ways, (1) genetically and (2) phenotypically on their behavioural responses to specific tests of temperament. We then intended to elucidate whether the behavioural and the physiological responses to a stressor are correlated.

A group of 160 adult Merino sheep of unknown temperament was genotyped for SNP rs107856856 to select two groups of sheep that carried either the genotype A/A (calm genotype, n = 9) or G/G (nervous genotype, n = 9). The same sheep were exposed to a standardised behavioural test and the responses were used to classify the sheep as either calm (the sheep with the lowest scores during an isolation box test, n = 9) or nervous (the sheep with the highest scores during an isolation box test, n = 9). The behavioural and physiological responses (T_c_, cortisol, prolactin, DHEA, and BDNF) of those sheep when exposed to several stressful situations, were then compared between the two genotype groups and the two phenotype groups of behavioural response.

## Results

### Genotyping of SNP rs107856856 in 160 sheep

Among the 160 sheep that were genotyped for SNP rs107856856, nine sheep carried a genotype (A/A) that is associated with calm temperament, and 97 sheep carried a genotype (G/G) that is associated with nervous temperament (Supplementary file 1). The remaining 54 sheep carried the heterozygous genotype (A/G).

### Behavioural responses to stress in groups classified according to genotype or behavioural phenotype

**Figure 1:**
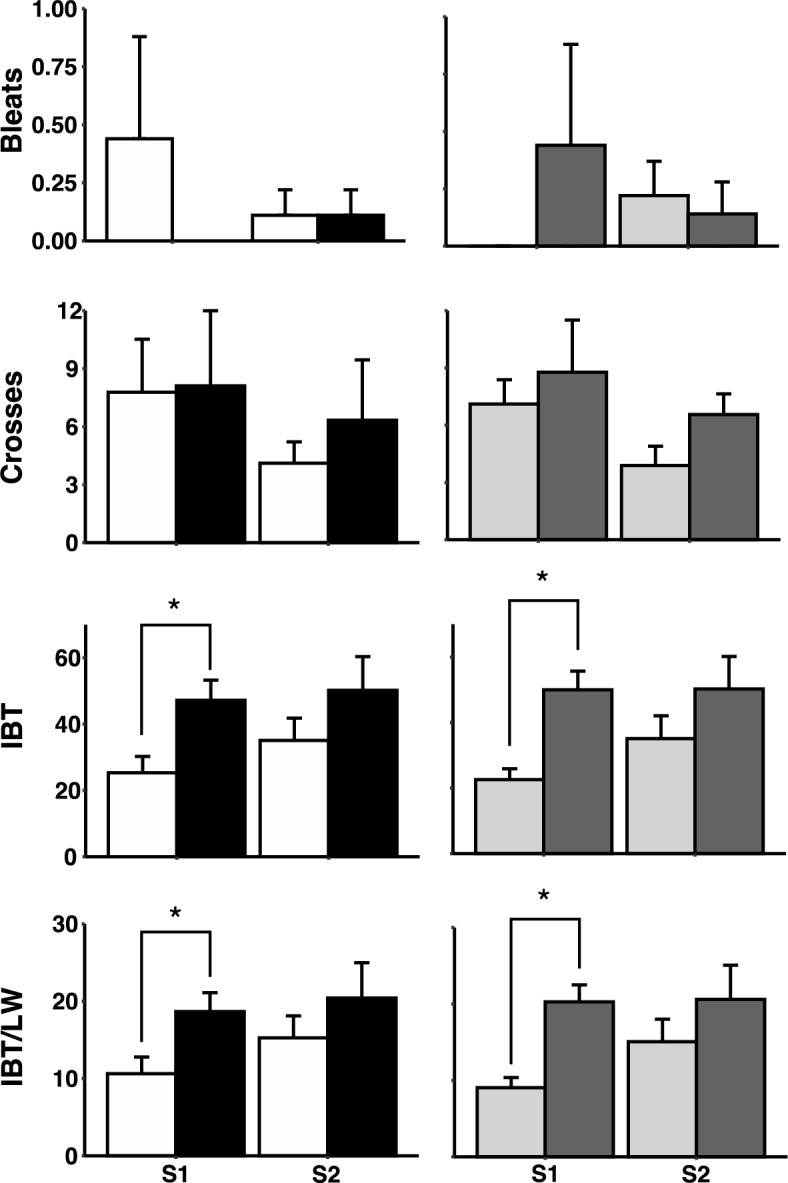
Scores for number of bleats, and number of crosses during the arena test, IBT, and IBT-LWT (standardised to the body weight of each animal) in sheep with the Calm A/A (white bar) or the Nervous G/G (black bar) genotype for SNP rs107856856, and in sheep that were phenotypically classified as low responder (grey bar) or high responder (dark grey bar) in two sessions (S1 = 1st session, S2 = 2nd session) of the behavioural tests. Data are shown as mean ± SE. * indicates a significant difference *P* < 0.05.

Seven of sheep carrying genotype A/A were classified as low responder, while two sheep carrying genotype A/A were classified high responder (Supplementary Table 1). Likewise, seven sheep carrying genotype G/G were classified as high responder, while two sheep carrying genotype G/G were classified as low responder (Supplementary Table 1). Genotype had a significant effect on the IBT in S1 (Fig. 1), with the sheep carrying genotype G/G scoring higher than the sheep carrying genotype A/A (P = 0.01; Fig. 1). But genotype had no effect on the behavioural responses during the arena test (bleats or crosses, P = 0.35 and 0.91 respectively). There was no effect of genotype on any of the behavioural responses during S2. The results from S1 were used to classify the sheep as either high or low responders, and analysis confirmed that the high-responding sheep scored higher than the low-responding sheep in S1 (P = 0.001; Fig. 1). In S2, there was no difference in behavioural responses between the low and high responding sheep (P > 0.05, Fig. 1).

### Physiological responses to stress in groups classified according to genotype or behavioural phenotype

**Figure 2:**
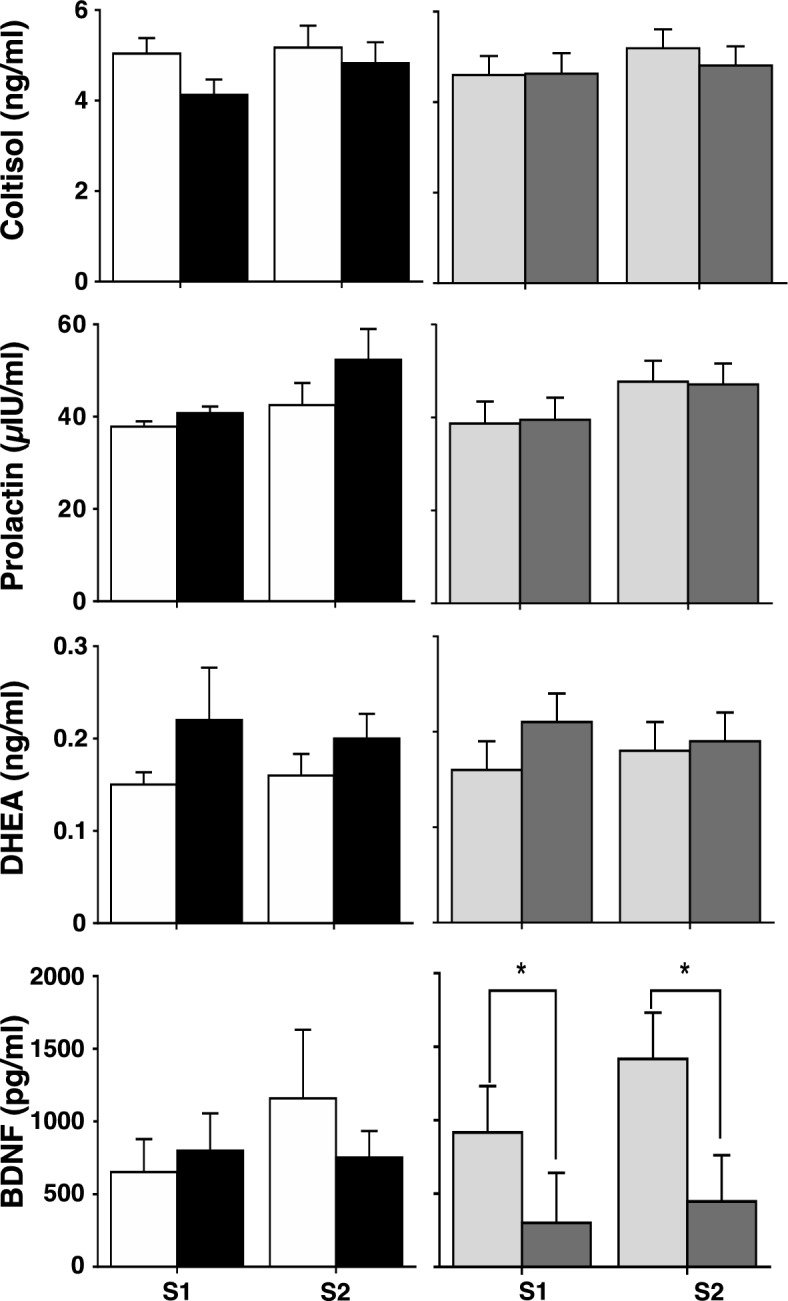
Plasma concentrations, at the end of each session (S1 = 1st session, S2 = 2nd session) of the behavioural tests, of cortisol (upper strip), prolactin (second strip), DHEA (third strip), and BDNF (lower strip) in sheep carrying the Calm A/A (white bar) or the Nervous G/G (black bar) genotype for SNP rs107856856, and in sheep that were phenotypically classified as low responder (grey bar) or of high responder (dark grey bar) in two sessions (S1 = 1st session, S2 = 2nd sesision) of the behavioural tests. Data are shown as mean ± SE. * indicates a statistical difference *P* < 0.05.

There was no effect of genotype on the plasma concentration of cortisol, prolactin, DHEA, or BDNF that were measured after S1 or S2 (P = 0.16, P = 0.15, P = 0.12 and P = 0.64 respectively; Fig. 2) or any effect of session (P = 0.36, P = 0.08, P = 0.90, and P = 0.43 respectively; Fig. 2). When the sheep were grouped by behavioural phenotype, the sheep from the low-responder group had a higher plasma concentration of BDNF than did the sheep in the high-responder group (P = 0.02; Fig. 2). There was no effect of behavioural phenotype on the other components of the blood (cortisol P = 0.96, prolactin P = 0.51, and DHEA P = 0.49; Fig. 2). Similarly, there was no effect of session on the plasma concentration of cortisol, prolactin, DHEA, or BDNF (P = 0.43, P = 0.19, P = 0.59 and P = 0.33 respectively; Fig. 2).

There was no effect of genotype on blood oxidative stress (P = 0.74), but session (P < 0.001) and time (before/after each test) (P = 0.01) had a significant effect on the level of oxidative stress (Supplementary Table 2). There was no interaction between genotype and session (P = 0.29), genotype and time (before/after each test) (P = 0.69), or genotype, session, and behavioural test (P = 0.88). The interaction between session and time (before/after each test) was significant (P = 0.01).

There was no effect of behavioural phenotype on oxidative stress (P = 0.42; Supplementary Table 3), but there was an effect of session (P < 0.001; Supplementary Table 3) and time (P = 0.01). There was no interaction between behavioural phenotype and session (P = 0.99), behavioural phenotype and time (before/after each test) (P = 0.86), or three-way interaction between behavioural phenotype, session, and time (before/after each test) (P = 0.73). The interaction between session and time (before/after each test) was significant (P = 0.01).

**Figure 3:**
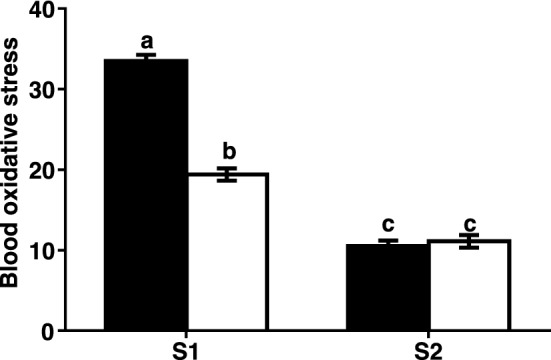
Level of blood oxidative stress of all the experimental sheep (there was no effect of either genotype or phenotype) before (white bar) and after (black bar) each of the behavioural tests during the two sessions (S1 = 1st session; S2 = 2nd session). Oxidative stress was calculated as the ratio of oxidised thiol groups on albumin to the non-oxidised groups (mean ± SE). Means with different superscripts are statistically different at P < 0.05.

Without considering the impact of genotype or behavioural phenotype, the level of blood oxidative stress was significantly higher before the behavioural test than after the test in S1 (P < 0.01) but did not change during S2 (P = 0.84, Fig. 3). The level of blood oxidative stress was significantly higher in S1 than it was in S2 either before or after the behavioural test (P < 0.01 and P = 0.01 respectively), even though it decreased significantly from before S1 to after S1.

### Stress induced hyperthermia in groups classified according to genotype or behavioural phenotype

**Figure 4:**
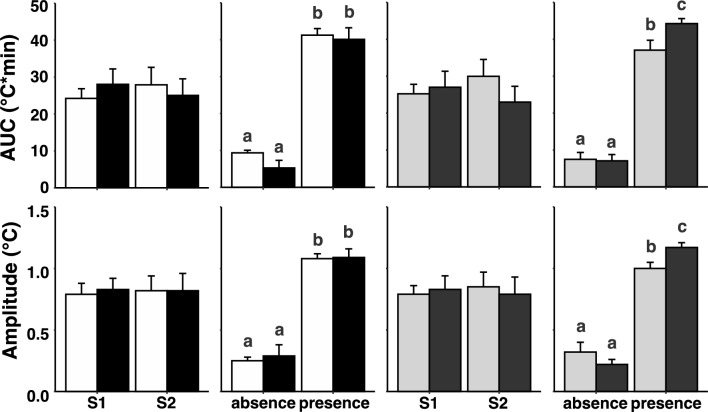
Area under the curve (AUC; top strip), and amplitude (lower strip) of the stress induced hyperthermia (mean ± SE) in response to behavioural testing during the two sessions (S1 = 1st session; S2 = 2nd session),and in response to the presence or absence of dogs (absence = absence of dogs; presence = presence of dogs) in sheep carrying the Calm A/A (white bar) or the Nervous G/G (black bar) genotypes for SNP rs107856856, and in sheep that were phenotypically classified as either low responder (grey bar) or high responder (dark grey bar) Bars show mean ± SE. Means with different superscripts within a graph indicate statistical difference at P < 0.05.

There was no effect of genotype or phenotype on the AUC or amplitude of the SIH response to either S1 or S2. After the sessions, when the sheep returned to their home paddock, the AUC and the amplitude of T_c_ was higher when the dogs were present (after S2) than when the sheep moved spontaneously (after S1), regardless of genotype or phenotype (P < 0.001, Fig. 4). There was no effect of genotype on the AUC of T_c_ or the amplitude of the T_c_ response after the testing sessions, but there was a difference when the sheep were classified by phenotype. When the sheep were grouped by behavioural phenotype, after S2, when the dogs were present, the AUC of T_c_ and the amplitude of the T_c_ response was significantly higher in the high responders than it was in the low responders (P = 0.02) (Fig. 4).

### Correlation between behavioural and physiological responses

**Figure 5:**
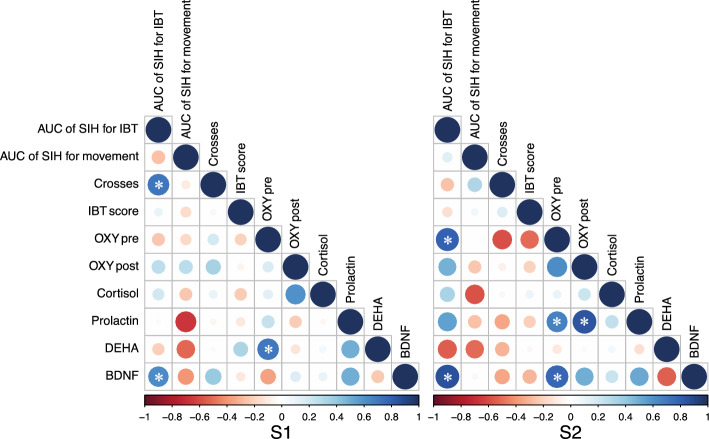
Correlation between each of the behavioural and physiological responses to stress that were measured in each of the two sessions of behavioural tests of all animals regardless of groups (genotype or phenotype) except for animals that had a missing variable. *AUC* the area under the curve of the stress induced hyperthermia (SIH), *IBT* isolation box test, *OXYPre* the blood oxidative stress before each behavioural test, *OXYPost* the blood oxidative stress after each behavioural test, *DHEA* dehydroepiandrosterone, *BDNF* brain derived neurotrophic factor, *S1* the first session of behavioural tests, *S2* the second session of behavioural tests. The size of circle and the colour scale indicate the coefficient values of each combination (as given in the legend below the figure). * indicates a significant correlation at *P* < 0.05.

In first session of behaviour tests (S1), the number of crosses was correlated with the AUC of SIH during the IBT (r = 0.71, P = 0.02) (Fig. 5). There was no strong correlation between any of the behavioural responses (crosses or IBT score) and any of the physiological responses in the first session of behaviour tests (S1). Some of the physiological responses correlated with each other: the AUC of SIH during IBT was correlated to BDNF (r = 0.63, P = 0.05) (Fig. 5) and oxidative stress before the testing (OXY pre) was significantly correlated to DHEA (r = 0.71, P = 0.02) (Fig. 5).

In the second session of behaviour tests (S2), there was no strong correlation between the behavioural responses and the physiological responses. Some of the physiological responses correlated with each other: the AUC of SIH in the IBT was correlated to OXY Pre (r = 0.80, P = 0.01) (Fig. 5) and BDNF (r = 0.85, P < 0.01) (Fig. 5). Moreover, OXY Pre was significantly correlated to BDNF (r = 0.78, P < 0.01) (Fig. 5), while OXY Post was significantly correlated to prolactin (r = 0.85, P < 0.01) (Fig. 5).

### Activity score in groups classified according to genotype or behavioural phenotype

**Figure 6:**
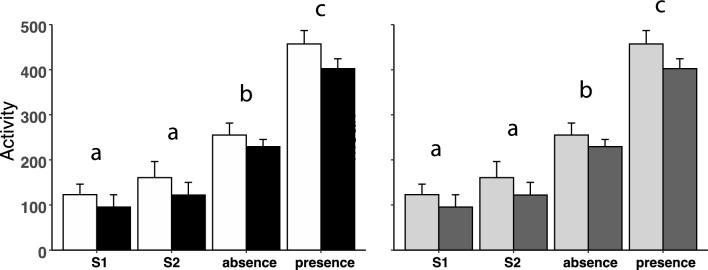
The activity score in sheep carrying the Calm A/A (white bar) or the Nervous G/G genotypes (black bar) for SNP rs107856856, and in sheep that were phenotypically classified as low responder phenotype (grey bar) or of high responder phenotype (dark grey bar) in two sessions (S1 = 1st session; S2 = 2nd session), and in response to the presence or absence of dogs (absence = absence of dogs; presence = presence of dogs). Data are shown as mean ± SE. There was no effect of either genotype pr phenotype, and so means with different superscripts within a graph indicate a statistical difference between periods (S1, S2, absence, and presence) at P < 0.05.

There was no significant effect of genotype or behavioural phenotype on the activity score of the sheep during either S1 or S2, or during the return to their home paddock (Fig. 6). As might be expected, the activity score was significantly higher during the return to the home paddock than it was during either S1 or S2, because the animals walked back to their home paddock. The activity score was higher in the presence of the dogs than when the sheep moved spontaneously back to their home paddock (P < 0.001) (Fig. 6).

### Circadian rhythm of core body temperature in groups classified according to genotype or behavioural phenotype

**Figure 7:**
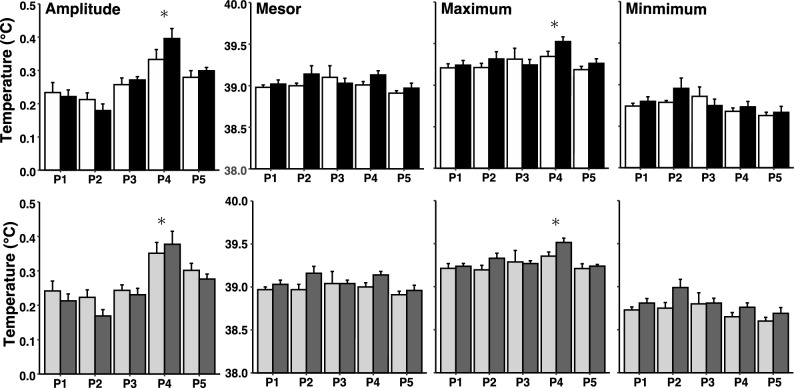
Characteristics of the circadian rhythm of body temperature in sheep carrying the Calm A/A (white bar) or the Nervous G/G (black bar) genotypes for SNP rs107856856, and in sheep that were phenotypically classified as either low responder (grey bar) or high responder (dark grey bar) (mean ± SE, n = 7). From left to right, the panels show amplitude, mesor, cosinor minimum, cosinor maximum over three days on five occasions from prior to the first behavioral test day (Period 1; P1), the three days of immediately after the first behavioral test day (Period 2; P2), the three days immediately after that (Period 3; P3), the three days immediately after the second behavioral test day (Period 4 ; P4), and finally the 3 days after that (Period 5 ; P5), as illustrated on Fig. 8. Bars show mean ± SE. * indicates P < 0.001 in the pairwise *t* test to P1.

There was no significant effect of genotype or phenotype on any of the parameters of the CRT (Fig. 7). The amplitude of the CRT during Period 4 (P4) was higher than it was during Period 1 (P1) (P < 0.001, Fig. 7). The cosinor maximum of the CRT in P4 was higher than it was during P1 (P < 0.001, Fig. 7). The mesor and cosinor minimum were not different between any of the periods.

## Discussion

The primary hypothesis that adult sheep with different genotypes of SNP rs107856856 would have different behavioural and physiological responses to stressors was partially supported.

Sheep from the Calm A/A group had a significantly lower IBT score than sheep from the Nervous G/G group, which suggests that the genotype did indeed impact on the behavioural temperament of the sheep. But there were no differences in the number of crosses or bleats between these groups, or in any of the physiological responses to stressors, including T_c_, cortisol, prolactin, DHEA, BDNF, or oxidative stress. Interestingly, the behavioural phenotype was related to the plasma concentrations of BDNF, with the low responders having significantly higher BDNF than the high responders after both S1 and S2. Similarly, the behavioural phenotype was related to the size of the SIH produced in the presence of dogs. Combined, all these findings suggest that the presence of rs107856856, that has been shown to be associated with behavioural traits in young sheep, has limited effects on the behavioural and physiological responses to stressors in adult sheep, but that the behavioural phenotype is associated with some aspects on the physiology.

Our results suggest that, in adult sheep, SNP rs107856856 can be used as a predictor of the behavioural response to social isolation as a stressor, but not to human contact as a stressor. The absence of an association between the SNP in TPH2 and the response to a human suggests either that SNP rs107856856 is only linked to the behavioural responses to isolation, or that habituation of our sheep to human contact over time had attenuated their reactivity to humans. Previously, SNP rs107856856 was identified as a predictor for temperament by measuring the distribution of the genotypes in two phenotypic lines of sheep that had been selectively bred for calm temperament or for nervous temperament for several generations^9,10,31,32^. In that previous study, two lines of sheep were selected on the combination of behavioural responses to human contact and to social isolation at 12 weeks of age. That selection led to divergence in both the number of crosses and the IBT score in the two lines, and those two lines were shown to have genetic differences in SNP rs107856856^9,10,31,32^. In the present study, the genotype of rs107856856 was used to group the sheep, and then the behavioural responses were compared between the groups. Sheep from the Calm A/A group had a significantly lower IBT score, but similar crosses, compared to sheep from the Nervous G/G group, suggesting that rs107856856 is related to the behavioural response to the stressor of social isolation but not to the stressor of human contact.

It seems that our results do not support the proposal that SNP rs107856856 can be used as a genetic predictor of temperament in older sheep^10^. It is probable that the timing of the behavioural testing influenced the association, or not, between the SNP and sheep behaviour^10^. In the previous study, the behavioural tests that were used to assess temperament were carried out around the time of weaning (12 to 14 weeks old) when sheep are very responsive to stressors^10^. It must be noted that the nature of those experiences (positive or negative) will likely affect the response of the sheep to humans^33^ and so responses may differ depending on the nature of prior experiences. It is likely that our animals might have gradually adapted to the stressor of handling and human presence, which could have reduced the perceived threat of humans. For example, cattle raised indoors are more docile than those raised on pasture, likely because those raised indoors have more interaction with humans^34^. Similarly, in sheep, repeated handling reduced the behavioural and physiological responses to human contact^35^. In the present study, the adult sheep would have experienced multiple episodes of management by humans, including yearly shearing, which would have familiarised them to humans, and likely resulted in the loss of effect of rs107856856 on the behavioural response to human contact (crosses and bleats). Further, while we classified the high and low responders according to their IBT score during S1, we saw no difference between the phenotypes at S2. The reason of the lack of the difference in IBT between calm and nervous, and low and high responders, might have been habituation to the test situation.

In the present study, we also assessed the physiological responses of the Calm A/A and Nervous G/G groups at the end of each session of behavioural testing. The lack of difference between the genotypes in cortisol, prolactin, DHEA, BDNF, and OXY responses to the behavioural testing indicates that the genotype of SNP rs107856856 does not influence these physiological responses to stressors in sheep. A first interpretation of those outcomes would be that there is no concordance between the behavioural and physiological responses to the stressors that we applied in the current work; however, we must keep in mind that in the current work SNP rs107856856 was associated with only some aspects of the behavioural responses to those stressors.

The findings from this current work contrast with previous work in rhesus monkeys that identified 17 SNPs in TPH2 gene and found that variation of one of those 17 SNPs resulted in different activity of the HPA axis in rhesus monkeys^22^. Thus, the lack of difference in physiological responses to stressors between the two groups of sheep with different genotypes of SNP rs107856856 could be attributed to the selection of a singular SNP, which might not fully predict all of the phenotypic variations among individuals^36^. By the time that our sheep reached the adult stage, repeated human contact during husbandry practices may have blunted the physiological responses to human contact, resulting in the absence of an association between rs107856856 and the physiological responses to tests that assess the effect of the human as a stressor. In a previous study, like with the behavioural response, repeated human contact has been shown to reduce the physiological responses to human contact in sheep^35^. Whatever the factors are that may affect the association between rs107856856 and temperament, SNP rs107856856 may not induce enough variation in the serotonin pathways in our sheep to induce difference in the physiological response to stressors in adult sheep.

Interestingly, BDNF was positively correlated with the AUC of SIH during the IBT, suggesting that BDNF could be involved in the thermal response, or that the thermal response could induce the expression of BDNF. Mice, that have reduced social interaction after experiencing social defeat, showed high BDNF levels in the nucleus accumbens and elevated hyperthermia during stress induced by social defeat^37^. Moreover, it has been suggested that BDNF positive neurons in the paraventricular nucleus have a role in the control of energy expenditure via brown adipose tissue thermogenesis^38,39^. Whether there are physiologically relevant levels of brown adipose tissue in adult sheep, as there are in adult rodents, is equivocal^40,41^, and so BDNF neurons might impact on thermogenesis via that mechanism, or there might be another mechanism that underpins the involvement of BDNF in the development of SIH in the adult sheep. Further studies are required to elucidate the involvement of brown adipose tissue thermogenesis in the development of SIH in sheep.

Our results suggest that psychological stress in sheep can induce oxidative stress, as measured as thiol oxidation of albumin in the blood, however that response was not affected by either genotype of phenotype. Oxidative stress occurred highest while the sheep were waiting to undergo the behaviour tests in S1; were lowered immediately after that test, and were lower then again prior to S2 having showed no change during S2. Oxidative stress is a measure of the production of residual free radicals present after clearance by antioxidant reserves in the body which has mostly been studied in relation to physical exercise in athletes^42^. The present study is the first time that the oximetric method^42^ has been used to measure oxidative stress in sheep. It is interesting that the oxidative stress was significantly higher prior to the first exposure to a behavioural test, and was then lower after the test, and at the next testing session. This finding is contradictory to the previous suggestions that psychological stress is presumed to induce oxidative stress, and our assumption that exposure to the tests themselves would induce a good level of psychological stress. For example, in mice and rats, the psychological or emotional distress that is induced by human handing or cutting off whiskers increased oxidative stress in the brain, heart, kidney, and liver^43–46^. Similarly, psychological stress resulted in an increase in oxidative stress in humans^47^. Although it is unclear why oxidative stress in the sheep decreased after being exposed to a stressful situation, the large decrease in oxidative stress that was measured as oxidised albumin in blood samples may be used as an indicator for stressed mental states related to human contact and isolation. It is possible that our sheep might have been stressed while they were waiting their turn for the behavioural testing and blood sampling in S1, but then habituated to those procedures after that first exposure to the test regime. Sheep have the cognitive ability to evaluate events that are happening in their environment according to the predictability of those events, which in turn modulates their emotional and behavioural responses to those events^48–50^. Our experimental sheep might have recognised the testing place and/or experimenter, which predicted what would happen to them in the second session. Therefore, this might have signified to the sheep that their situation will be a benign experience. In contrast, the first session was an entirely novel situation. That is probably why in the second session of behavioural tests, there was no difference in the oxidative stress before and after the behavioural tests. These findings suggest that oxidative stress could be used as an indicator of the perception of stressful events and the capacity of an animal to habituate to repeated exposure to the eliciting event.

However, the oxidative stress was not affected by the genotype for TPH2, which is surprising since a reduction of 5-HT, achieved by using an inhibitor of tryptophan hydroxylase, has been shown to decrease antioxidant status in rat brain, while exogenous supplementation of the precursors of serotonin increased serotonin levels in the brain and prevented an increase in oxidative stress similar to that induced by depression^51,52^. In the present study, it is possible that the SNP in the TPH2 gene did not induce a large difference in the efficiency of the TPH2 enzyme, and therefore there was not a difference in serotonin levels in the brain of our sheep. It would be informative to test the impact of a serotonin inhibitor, such as p-chlorophenylalanine, on brain serotonin and changes in oxidative stress to confirm the relationship between serotonin and oxidative stress in sheep.

Our data suggests that SIH might reflect the emotional reactivity of individual sheep. Sheep that responded strongly in the IBT test (and were therefore assigned to the “high responder” phenotype) had a stronger SIH response to the dog exposure than did those sheep classified as “low responders”. However, an SIH response was only observed after exposure to a dog and not after the arena or the isolation tests while both tests are meant to measure the emotional reactivity of sheep^53^. Therefore, it seems that the SIH might not be a very sensitive indicator of emotional response but rather an indicator of high level of fear. Even in presence of a dog, the thermogenic pathway in high responders seemed to be activated more by psychological stress than it was in the low responders. On the other hand, the SNP in TPH2 didn’t affect the SIH response, even though it affected the IBT score. As suggested by the lack of impact of the SNP on other physiological markers of the stress response, discussed above, the serotonergic pathway might not be the only neural mechanism that is involved in the determination of emotional reactivity and the activation of SIH. For example, SNPs in the dopamine receptor and cytochrome P450 17α-hydroxylase/17,29-lyase are part of the dopaminergic pathway and have been associated with temperament^9^, and that pathway is involved in the modulation of stress induced hyperthermia in mole rat and rat^54,55^. Similarly, the oxytocinergic pathway has been associated with temperament in sheep^10^ and oxytocin null mice that had an oxytocin deficient had a larger SIH in response to psychological stressors than did wild type mice^56^. Further investigations are required to uncover the relationship between the genotypes that are related to temperament and the thermogenic response to stress. These other pathways might be involved in the development of SIH and might also differentiate between high and low responders. Although further studies are required to elucidate the potential pathways involved in the differentiation of the physiological response, SIH is already considered as a reliable indicator of psychological stress^57^ in rats, and so could indicate an animal’s emotional reactivity.

In addition, our results suggest that the amplitude of the CRT could be a long-lasting indicator of the psychological stress that an animal has experienced. Although there was no difference in the amplitude of the CRT between sheep with contrasting temperament determined by either genotype or behavioural phenotype, the amplitude was higher for several days after exposure to dogs (period 4 after session 2) than it was before the testing sessions (period 1). The mechanism(s) that control the amplitude of the CRT is/are unknown^58^. However, it is known that the amplitude of the CRT changes for a few days in response to a challenge to homeostasis^58^. For example, a decreased energy intake increases the amplitude of the CRT^59,60^. Changes in the amplitude of the CRT are observed within a day after the modification of energy intake, suggests that a change of amplitude is sensitive, immediate, and integrative^58^; such as in our study. Similarly, water balance and reproductive status can also affect the amplitude of the CRT^58^. The possibility that psychological stress has a long-term impact (lasting for a few days) on the amplitude of CRT suggests that studying mechanisms of the relationship between psychological stress and control of the amplitude of CRT could prove fruitful.

In the present study, the number of animals possibly limited our power to detect an impact of genotype or phenotype on the physiological responses, or the correlations between the behavioural and physiological responses. It is possible that a larger number of animals would have led to stronger correlations and significant effects of both genotype and phenotype.

Overall, the present study has helped to identify that albumin thiol oxidation, BDNF, the SIH response, and the amplitude of the CRT could be relevant indicators of mental stress in Merino sheep. It was somewhat surprising that SNP rs107856856 had limited effects on the temperament phenotype in adult sheep. However, our study suggests that the pathways between the perception of stressors and the physiological and behavioural responses that are evoked by the stressors involves several central pathways. We propose that a genome-wide association study that compares different phenotypes and a transcriptome study in specific areas of the brain that are involved in the formation of emotion would help us to understand these pathways.

## Materials and methods

This experiment was approved by the Animal Ethics Committee of The University of Western Australia (Approval RA/3/100/1691) and was carried out in accordance with the ARRIVE guidelines and the Australian Code of Practice for the Care and Use of Animals for Scientific Purpose (8th Edition, 2013).

### Experimental animals and design

**Figure 8:**
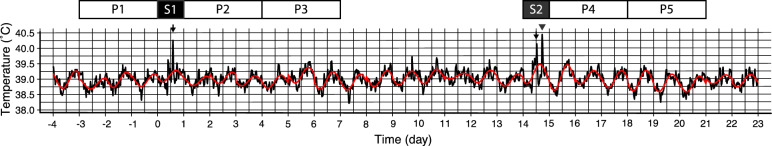
Experimental timeline and representative profile of core body temperature in one sheep for the duration of the experiment. The plot shows the original record of 5-min recordings of T_c_ (black line), and fitted cosinor for each 24 h (red line). The black numbered boxes above the plot indicate the days when behavioural phenotype was assessed. The stress induced hyperthermia (SIH) that was associated with each stressor is indicated by arrows (behavioral test) and triangle (exposure to dog). Each period of three days when the characteristics of the circadian rhythm of core body temperature (CRT) was analysed is indicated by a numbered open box above the trace (P1 etc.)

A total of 160 castrated Merino sheep (3-year-old) from a flock kept at The University of Western Australia (UWA) farm that had never been studied for temperament were used in this study. The sheep were genotyped for SNP 107856856, an SNP that has been identified as a genetic marker for temperament^10^. From the 160 experimental sheep that were tested, nine sheep carrying the genotype A/A (calm genotype), and another nine sheep carrying the genotype G/G (nervous genotype) were selected for further study. A temperature and activity logger was surgically implanted intra-abdominally into those sheep to measure core body temperature and activity throughout the experiment. Those 18 sheep were exposed to two sessions of behavioural tests within a two-week interval (hereafter called S1 and S2) (Fig. 8). After S1, the 18 sheep were classified phenotypically into two groups; a low-responder group that consisted of the nine sheep that had the lowest scores during the first isolation box test (see below), and a high- responder group that consisted of the nine sheep that had the highest scores during the first isolation box test. The two genotype groups, and the two phenotype groups, were compared for differences in behavioural and physiological responses to S1 and S2.

### Genotyping for SNP 107856856

Whole blood from the 160 sheep was sampled from a jugular vein using a vacutainer tube containing Ethylenediaminetetraacetic acid (EDTA) (Greiner Bio-One, Australia). Genomic DNA was extracted using a DNeasy Blood and Tissue Kit (69506, Qiagen, Hilden, Germany) following manufacturer instructions. The concentration of genomic DNA was measured with a Nanodrop spectrophotometer (ThermoFisher, Scoresby, Australia), and the integrity of the genomic DNA was assessed by agarose gel electrophoresis. SNP rs107856856 was genotyped using an Agena Bioscience Mass ARRAY as described previously^10^.

### Behavioural tests

The behavioural responses of the sheep were assessed using an open-field arena test and an isolation box test (IBT)^53^. Each sheep was introduced into a test arena (7 × 3.3 m) that was divided by lines on the floor into four sectors. Each sheep remained in the arena for 3 min. A motionless human who was unfamiliar to the experimental sheep stood at one end of the arena, opposite to the entrance, in front of a small pen that contained three sheep from the same flock as the test sheep. The behavioural response of each sheep was quantified by counting the number of bleats and the number of sector crosses that it made in those 3 min.

Within 2 min after the completion of the arena test, each sheep was placed into an isolation box (1.5 × 1.5 × 0.75 m) for 1 min. During the IBT, an agitation metre on the side of the box recorded vibrations from the box that were caused by movement and vocalisations. The agitation metre was calibrated prior to each use with an electrotonic “sheep” (27 kg) that produced three standardised levels of movement^31^. The agitation value from the IBT was normalised for live weight (LW) by dividing the raw IBT score by the live weight of the animal and multiplying by the weight of the device that was used to calibrate the isolation box (27 kg). The normalised IBT score from S1 was used to classify each experimental sheep into the low-responder or the high-responder group.

Each session of behavioural testing was conducted in an enclosed shed and the sheep were moved to the shed at least 2 h before the start of testing. At the end of S1, the gates on the route back to the home paddock of the sheep were left open, and the sheep walked spontaneously back to their home paddock that was located 300 m away from the testing shed. After S2, the sheep were herded to their home paddock by two dogs and a farmhand. The pace of movement back to the home paddock after S2 was kept as close as possible to the spontaneous movement after S1.

### Measurement of the physiological response to stressors

#### Measurement of core body temperature and activity

The core body temperature (T_c_) and activity of each sheep was measured every 5 min using an intra-abdominal logger that had been implanted four weeks before S1. The loggers (Bryn O Morgan Industries, Perth, WA) were covered in inert wax (Sasol EXP987, Johannesburg, South Africa). The loggers were equipped with a 3D accelerometer and activity was recorded every 5 min by adding the average activity from each of the three axes together. We used that composite index as activity score. The temperature resolution of the loggers was 0.02 °C. The loggers were calibrated against a certified precision platinum thermometer (Center 376, Center Technology Corp., Taipai, Taiwan; calibrated by WIKA Australia, Rydalmere, certified by the National Association of Testing Authorities, Australia) between 33 and 42 °C before and after their time in the animals. The loggers recorded T_c_ every 5 min to an accuracy of better than 0.05 °C. The loggers were sterilised by immersion in a solution of 0.5% chlorhexidine (Chlorhex C, Jurox, Rutherford, NSW) and 70% ethanol for at least 24 h before implantation.

Each sheep was anesthetised by intramuscular injection of a mixture of ketamine (6.3 mg kg^−1^), xylazine (0.63 mg kg^−1^) and butorphanol (0.03 mg kg^−1^). A 10 cm incision was made about 10 cm behind the last rib. The overlying muscle layers were dissected down to the peritoneal wall, and small incision was made, the datalogger was inserted, and tethered to the peritoneal wall. Deviations in rumen temperature that can be caused by drinking or from the heat of fermentation do not impact on the temperature measured at that site^61^, except when, with a time lag, those changes impact on the temperature of the body more generally^62^. A single silk suture was used to anchor the datalogger to the peritoneal wall. The wall and muscle layers were sutured using Vicryl. The animals received a post-operative course of analgesic (4 mg kg^−1^ Carprofen) medication and were given two weeks to recover. The loggers were retrieved from the abdominal cavity at the time of slaughter.

#### Measurement of cortisol, prolactin, DHEA, and BDNF

Before and after the behavioural tests in each session, blood was sampled from a jugular vein using a vacutainer tube that contained EDTA (Greiner Vacuette, Australia) within 1 min. A total of four blood samples was collected from each sheep during the whole study. The samples were kept on ice until the plasma was separated by centrifugation at 2000*g*, at 4 °C, for 10 min. Plasma was then stored at − 20 °C until processing and analysis.

The plasma concentration of cortisol was measured in duplicate using an MP Biomedical I125 RIA cortisol Kit (#07-221106, Australian Bioresearch, Perth) following the manufacturer’s protocol and reagents. The limit of detection was 0.25 ng ml^−1^ and the intraassay variability was 6.5% (1.4 ng ml^−1^) and 1.8% (3.2 ng ml^−1^).

The plasma concentration of prolactin was measured in duplicate using a sheep ELISA kit (MBS702553, MyBioSource, San Diego, CA, USA). The limit of detection was 5 µIU ml^−1^ and the intraassay coefficient of variation was less than 15%.

The plasma concentration of DHEA was measured in duplicate with a sheep ELISA kit (MBS458844, MyBioSource, SanDiego, CA, USA). The limit of detection was 0.045 ng ml^−1^ and the intraassay coefficient of variation was less than 10%.

The plasma concentration of BDNF was measured in in duplicate with a sheep ELISA kit (MBS734286, MyBioSource, SanDiego, CA, USA). The limit of detection was 10 pg ml^−1^ and the intraassay coefficient of variation was less than 10%. The results from the ELISA were calculated using a four parameters logistic (4PL) regression model using Assayfit Pro (AssayCloud, Nijmegen, The Netherlands).

#### Measurement of oxidative stress

The blood level of oxidative stress was measured using an oximetric method that quantified the thiol S–H bonds present on the albumin^42^. Around 30–40 µl of blood was pipetted onto an oximetric blood spot card (PerkinElmer 226 protein-saver-5-spot cards) that contained a trapping agent consisting of a solution of methoxy polyethylene glycol 2000 dry powder (JenKem Technology, USA) in 40 mM imidazole (Sigma). The cards were immediately stored in a desiccator until processing.

To analyse the samples, a 4.5 mm hole was punched through the centre of each blood spot and placed into a well on a 96 well plate. The disks were eluted with 100 µl of 20 mM phosphate buffer at room temperature on a plate mixer for 2 h. An aliquot of 40 µl of eluted blood was then added to a solution of cibacron blue and incubated at room temperature for 10 min. After a short centrifugation, the pellet was washed with 100 µl of 20 mM phosphate buffer and centrifuged again to remove any unwanted components of whole blood. The bound albumin was eluted by adding 25 µl of 1.4 M sodium chloride, mixed and centrifuged to collect the supernatant that contained relatively purified albumin. The purified albumin solution was mixed with equal volumes of Laemmli 2× buffer and vortexed thoroughly. An aliquot of 20 µl was loaded onto a 16% polyacrylamide gel and the gel was run at 180 V, for 2 h. The gel was imaged on a ChemiDoc MP imaging system using a 5-min exposure. Total oxidation was expressed as the ratio of the intensity of the bands of oxidised albumin to the bands of non-oxidised albumin as quantified using Image J^63^.

### Data analysis

The area under the curve (AUC in °C × min) of the T_c_ response to a specific stressor was calculated by summing the difference between T_c_ and the running average of T_c_ for 12 h around that point (6 h prior and 6 h after) (T_s_). for 50 min after the time that the behavioural test started, or after the time point when the sheep started moving after the behavioural test had finished. The values were multiplied by five since T_c_ was measured every 5 min. The amplitude was defined as the maximum value of the difference between T_c_ and T_s_ in the period used for the calculation of the AUC of SIH during the 50 min.

The characteristics of the circadian rhythm of T_c_ (CRT) were calculated using a cosinor analysis on 24 h of data starting at 12 AM each day^58,64^. The analysis provided a mesor, amplitude, cosinor minimum, cosinor maximum, and the time of the acrophase for each sheep on each day. The characteristics of the CRT were compared between groups by taking an average for each sheep over three days on five occasions; the three days prior to S1 (Period 1; P1), the three days immediately after S1 (Period 2; P2), the three days prior to S2 (Period 3; P3), the 3 days immediately after S2 (Period 4; P4), and finally for the three days after P4 (Period 5; P5). An example of the profile of T_c_, fitted cosinor, the timing of the behavioural testing sessions, and the five periods during which the CRT was quantified is given in Fig. 8.

#### Analysis of behavioural and physiological responses to stress

The data were analysed to compare each variable between the two genotype groups and between the two phenotype groups using IBM SPSS Statistics for Windows (Version 22.0, IBM Corp., Armonk, NY: IBM Corp). A Shapiro–Wilk test and a Bartlett’s test were used to assess normality and homogeneity of the data. To normalise distributions, the data for blood oxidative stress was transformed using a log transformation, the variable prolactin was transformed using reciprocal transformation, and the variable DHEA was transformed using 1 divided by the square root transformation for analysis.

The main effect of genotype, session, and time (before/after each session), as well as their interactions, on each parameter was analysed using Multivariate General Linear Model analysis. Interactions were subsequently excluded when they were not significant.

The main effects of behavioural phenotype, session, and time (before/after each session) was analysed in the same way. For the level of blood oxidative stress, the main effect of genotype, session, and time (before/after each session), as well as the full interaction, and most of the two-factor interactions were initially included in the model, but then excluded in the final analysis because they were not significant. The interaction between session and time (before/after behavioural test) was retained. The main effect of behavioural phenotype, session, and time (before/after each session) was analysed in the same way. The effect of genotype on the AUC, amplitude, and duration of the SIH, was analysed using a Multivariate General Linear Model. The differences were analysed by pairwise comparison tests adjusted for multiple comparisons using the Bonferroni method.

For both S1 and S2, correlations between the AUC of SIH during IBT, the AUC of SIH during movement to paddock, cortisol, prolactin, DHEA, and BDNF, pre and post oxidative stress (OXY pre and OXY post), IBT score, and number of crosses during the arena test were analysed with Pearson correlation coefficients using R. Statistical significance of coefficient values were analysed using the “cor.test” function in the “psych” R package. These results were visualised using the “corrplot” R package. Any animal that had a missing variable was excluded from the correlation analysis.

Differences in the characteristics of the CRT over time were analysed using linear mixed-effects models followed by pairwise comparison tests adjusted for multiple comparisons using the false discovery rate method. The pattern of activity over the recording period was analysed using linear mixed-effects models followed by pairwise comparison tests adjusted for multiple comparisons using the false discovery rate method. Similarly, the effect of grouping using behavioural phenotype on the AUC, amplitude, duration of the SIH, and activity was analysed using Multivariate General Linear Model analysis as described above.

### Supplementary Information

Supplementary Tables.

Supplementary Information.

## Data Availability

The data analysed during the current study are available from the corresponding author on reasonable request.

## References

[1] Réale D, Reader SM, Sol D, McDougall PT, Dingemanse NJ (2007). Integrating animal temperament within ecology and evolution. Biol. Rev. Camb. Philos. Soc..

[2] Glenske K, Prinzenberg E-M, Brandt H, Gauly M, Erhardt G (2011). A chromosome-wide QTL study on BTA29 affecting temperament traits in German Angus beef cattle and mapping of DRD4. Animal.

[3] Glenske K, Brandt H, Prinzenberg E-M, Gauly M (2010). Verification of a QTL on BTA1 for temperament in German Simmental and German Angus calves (Short Communication). Arch. Anim. Breed..

[4] Chen Q (2020). Genome-wide association study identifies genomic loci associated with flight reaction in cattle. J. Anim. Breed. Genet..

[5] Paredes-Sánchez FA (2020). Novel genes involved in the genetic architecture of temperament in Brahman cattle. PLoS One.

[6] Dos Santos FC (2017). Identification of candidate genes for reactivity in Guzerat (*Bos*
*indicus*) cattle: A genome-wide association study. PLoS One.

[7] Boldt, C. R. A study of cattle disposition: Exploring QTL associated with temperament (Texas A&M university, Honors thesis, 2008).

[8] Hazard D (2014). Identification of QTLs for behavioral reactivity to social separation and humans in sheep using the OvineSNP50 BeadChip. BMC Genom..

[9] Qiu X, Ledger J, Zheng C, Martin GB, Blache D (2016). Associations between temperament and gene polymorphisms in the brain dopaminergic system and the adrenal gland of sheep. Physiol. Behav..

[10] Ding L (2021). Association between temperament related traits and single nucleotide polymorphisms in the serotonin and oxytocin systems in Merino sheep. Genes Brain Behav..

[11] Vanhonacker F, Verbeke W, Van Poucke E, Tuyttens FAM (2008). Do citizens and farmers interpret the concept of farm animal welfare differently?. Livest. Sci..

[12] Sevi A (2003). The effect of a gradual separation from the mother on later behavioral, immune and endocrine alterations in artificially reared lambs. Appl. Anim. Behav. Sci..

[13] Caroprese M (2010). Relationship between cortisol response to stress and behavior, immune profile, and production performance of dairy ewes. J. Dairy Sci..

[14] Pascucci T (2009). 5-Hydroxytryptophan rescues serotonin response to stress in prefrontal cortex of hyperphenylalaninaemic mice. Int. J. Neuropsychopharmacol..

[15] Basic D (2013). Changes in regional brain monoaminergic activity and temporary down-regulation in stress response from dietary supplementation with l-tryptophan in Atlantic cod (*Gadus*
*morhua*). Br. J. Nutr..

[16] Lepage O, Tottmar O, Winberg S (2002). Elevated dietary intake of l-tryptophan counteracts the stress-induced elevation of plasma cortisol in rainbow trout (*Oncorhynchus*
*mykiss*). J. Exp. Biol..

[17] Rosado B (2010). Blood concentrations of serotonin, cortisol and dehydroepiandrosterone in aggressive dogs. Appl. Anim. Behav. Sci..

[18] Popova NK, Naumenko VS (2019). Neuronal and behavioral plasticity: The role of serotonin and BDNF systems tandem. Expert Opin. Ther. Targets.

[19] Chen G-L, Miller GM (2012). Advances in tryptophan hydroxylase-2 gene expression regulation: New insights into serotonin-stress interaction and clinical implications. Am. J. Med. Genet. B Neuropsychiatr. Genet..

[20] Jacobsen JPR (2012). Deficient serotonin neurotransmission and depression-like serotonin biomarker alterations in tryptophan hydroxylase 2 (Tph2) loss-of-function mice. Mol. Psychiatry.

[21] Brivio P (2018). TPH2 deficiency influences neuroplastic mechanisms and alters the response to an acute stress in a sex specific manner. Front. Mol. Neurosci..

[22] Chen G-L, Novak MA, Hakim S, Xie Z, Miller GM (2006). Tryptophan hydroxylase-2 gene polymorphisms in rhesus monkeys: Association with hypothalamic-pituitary-adrenal axis function and in vitro gene expression. Mol. Psychiatry.

[23] Chen G-L (2010). The effect of rearing experience and TPH2 genotype on HPA axis function and aggression in rhesus monkeys: A retrospective analysis. Horm. Behav..

[24] Correia AS, Cardoso A, Vale N (2023). Oxidative stress in depression: The link with the stress response, neuroinflammation, serotonin, neurogenesis and synaptic plasticity. Antioxidants (Basel).

[25] Khanzode SD, Dakhale GN, Khanzode SS, Saoji A, Palasodkar R (2003). Oxidative damage and major depression: The potential antioxidant action of selective serotonin re-uptake inhibitors. Redox Rep..

[26] Nakamura K (2015). Neural circuit for psychological stress-induced hyperthermia. Temperature.

[27] Oka T (2015). Psychogenic fever: How psychological stress affects body temperature in the clinical population. Temperature.

[28] Nishitani N (2019). CRISPR/Cas9-mediated in vivo gene editing reveals that neuronal 5-HT1A receptors in the dorsal raphe nucleus contribute to body temperature regulation in mice. Brain Res..

[29] Hodges MR (2008). Defects in breathing and thermoregulation in mice with near-complete absence of central serotonin neurons. J. Neurosci..

[30] Olivier JDA (2008). Stress-induced hyperthermia and basal body temperature are mediated by different 5-HT1A receptor populations: A study in SERT knockout rats. Eur. J. Pharmacol..

[31] Blache D, Bickell SL (2010). Temperament and reproductive biology: Emotional reactivity and reproduction in sheep. Soc. Bras. Zootecnia.

[32] Murphy PM (1999). Maternal behaviour and rearing ability of Merino ewes can be improved by strategic feed supplementation during late pregnancy and selection for calm temperament.

[33] Hild S, Coulon M, Schroeer A, Andersen IL, Zanella AJ (2011). Gentle vs aversive handling of pregnant ewes: I. Maternal cortisol and behavior. Physiol. Behav..

[34] Le Neindre, P. *et al.* Genetics of maternal ability in cattle and sheep. In *Proceedings of the 6th World Congress on Genetics Applied to Livestock Production* (1998).

[35] Hargreaves AL, Hutson GD (1990). Some effects of repeated handling on stress responses in sheep. Appl. Anim. Behav. Sci..

[36] Wray NR (2013). Pitfalls of predicting complex traits from SNPs. Nat. Rev. Genet..

[37] Krishnan V (2007). Molecular adaptations underlying susceptibility and resistance to social defeat in brain reward regions. Cell.

[38] An JJ, Liao G-Y, Kinney CE, Sahibzada N, Xu B (2015). Discrete BDNF neurons in the paraventricular hypothalamus control feeding and energy expenditure. Cell Metab..

[39] Wang P (2020). A leptin–BDNF pathway regulating sympathetic innervation of adipose tissue. Nature.

[40] Pope M, Budge H, Symonds ME (2014). The developmental transition of ovine adipose tissue through early life. Acta Physiol..

[41] Henry BA, Blache D, Rao A, Clarke IJ, Maloney SK (2010). Disparate effects of feeding on core body and adipose tissue temperatures in animals selectively bred for Nervous or Calm temperament. Am. J. Physiol. Regul. Integr. Comp. Physiol..

[42] Lim L (2020). The relationship between intraoperative cerebral oximetry and postoperative delirium in patients undergoing off-pump coronary artery bypass graft surgery: A retrospective study. BMC Anesthesiol..

[43] Adachi S, Kawamura K, Takemoto K (1993). Oxidative damage of nuclear DNA in liver of rats exposed to psychological stress. Cancer Res..

[44] Wang L (2007). Psychological stress-induced oxidative stress as a model of sub-healthy condition and the effect of TCM. Evid. Based Complement. Alternat. Med..

[45] Rahman MM, Ichiyanagi T, Komiyama T, Sato S, Konishi T (2008). Effects of anthocyanins on psychological stress-induced oxidative stress and neurotransmitter status. J. Agric. Food Chem..

[46] Mousavi M-S (2019). Comparative evaluation of adolescent repeated psychological or physical stress effects on adult cognitive performance, oxidative stress, and heart rate in female rats. Stress.

[47] Sivonová M (2004). Oxidative stress in university students during examinations. Stress.

[48] Désiré L, Veissier I, Després G, Boissy A (2004). On the way to assess emotions in animals: Do lambs (*Ovis*
*aries*) evaluate an event through its suddenness, novelty, or unpredictability?. J. Comp. Psychol..

[49] Greiveldinger L, Veissier I, Boissy A (2007). Emotional experience in sheep: Predictability of a sudden event lowers subsequent emotional responses. Physiol. Behav..

[50] McAllister MJ (2019). Effects of psychological stress during exercise on markers of oxidative stress in young healthy, trained men. Physiol. Behav..

[51] Anderson G, Maes M (2014). Oxidative/nitrosative stress and immuno-inflammatory pathways in depression: Treatment implications. Curr. Pharm. Des..

[52] Muñoz-Castañeda JR (2006). Role of serotonin in cerebral oxidative stress in rats. Acta Neurobiol. Exp..

[53] Beausoleil NJ, Blache D, Stafford KJ, Mellor DJ, Noble ADL (2008). Exploring the basis of divergent selection for ‘temperament’ in domestic sheep. Appl. Anim. Behav. Sci..

[54] Brizuela M, Antipov A, Blessing WW, Ootsuka Y (2019). Activating dopamine D2 receptors reduces brown adipose tissue thermogenesis induced by psychological stress and by activation of the lateral habenula. Sci. Rep..

[55] Antipov A, Brizuela M, Blessing WW, Ootsuka Y (2020). Alpha2-adrenergic receptor agonists prevent emotional hyperthermia. Brain Res..

[56] Amico JA, Mantella RC, Vollmer RR, Li X (2004). Anxiety and stress responses in female oxytocin deficient mice. J. Neuroendocrinol..

[57] Vinkers CH (2009). Stress-induced hyperthermia is reduced by rapid-acting anxiolytic drugs independent of injection stress in rats. Pharmacol. Biochem. Behav..

[58] Maloney SK, Goh G, Fuller A, Vesterdorf K, Blache D (2019). Amplitude of the circadian rhythm of temperature in homeotherms. CAB Rev. Perspect. Agric. Vet. Sci. Nutr. Nat. Resour..

[59] Maloney SK, Meyer LCR, Blache D, Fuller A (2013). Energy intake and the circadian rhythm of core body temperature in sheep. Physiol. Rep..

[60] Goh GH, Mark PJ, Maloney SK (2016). Altered energy intake and the amplitude of the body temperature rhythm are associated with changes in phase, but not amplitude, of clock gene expression in the rat suprachiasmatic nucleus in vivo. Chronobiol. Int..

[61] Vesterdorf K, Beatty DT, Barnes A, Maloney SK (2022). Rumen temperature is a reliable proxy of core body temperature in sheep (*Ovis*
*aries*). Anim. Prod. Sci..

[62] Beatty DT, Barnes A, Taylor E, Maloney SK (2008). Do changes in feed intake or ambient temperature cause changes in cattle rumen temperature relative to core temperature?. J. Therm. Biol..

[63] Schneider CA, Rasband WS, Eliceiri KW (2012). NIH Image to ImageJ: 25 years of image analysis. Nat. Methods.

[64] Nelson W, Tong YL, Lee JK, Halberg F (1979). Methods for cosinor-rhythmometry. Chronobiologia.

