# Supplementary material for: Behavioural and physiological responses to stressors in sheep with temperament classified by genotype or phenotype
Source: Sci Rep. 2024 Apr 8;14:8147. doi: 10.1038/s41598-024-58959-y (PMC10999442; doi:10.1038/s41598-024-58959-y)
Supplement: Supplementary file 1 — Supplementary Tables. [file 41598_2024_58959_MOESM1_ESM.docx]

**Supplementary Table 1.** Concordance of the classification by genotype and by phenotype

|  | Genotype A/A | Genotype G/G |
| --- | --- | --- |
| Low responder | 7 | 2 |
| High responder | 2 | 7 |

**Supplementary Table 2.** Main effects and interactions between genotype, session, and time (before / after each testing session) on the level of oxidative stress in the blood of Merino sheep.

| Source | Type III Sum of Squares | df | Mean Square | F | P value |
| --- | --- | --- | --- | --- | --- |
| Genotype | 8.68 | 1 | 8.68 | 0.11 | 0.74 |
| Session | 3389.86 | 1 | 3389.86 | 44.61 | < 0.001 |
| Time | 585.03 | 1 | 585.03 | 7.7 | 0.01 |
| Genotype * Session | 85.33 | 1 | 85.33 | 1.12 | 0.29 |
| Genotype * Time | 12.15 | 1 | 12.15 | 0.16 | 0.69 |
| Session * Time | 618.24 | 1 | 618.24 | 8.14 | 0.01 |
| Genotype * Session * Time | 1.74 | 1 | 1.74 | 0.02 | 0.88 |

**Supplementary Table 3.** Main effects and interactions between behavioural phenotype, session, and time (before / after each testing session)on the level of oxidative stress in the blood of Merino sheep.

| Source | Type III Sum of Squares | df | Mean Square | F | P value |
| --- | --- | --- | --- | --- | --- |
| Phenotype | 51.1 | 1 | 51.1 | 0.67 | 0.42 |
| Session | 3307.7 | 1 | 3307.7 | 43.34 | < 0.001 |
| Time | 558.5 | 1 | 558.5 | 7.32 | 0.01 |
| Behavioural phenotype * Session | 0.02 | 1 | 0.02 | < 0.001 | 0.99 |
| Behavioural phenotype * Time | 2.40 | 1 | 2.40 | 0.03 | 0.86 |
| Session * Time | 672.36 | 1 | 672.36 | 8.81 | 0.01 |
| Behavioural phenotype * Session * Time | 8.9 | 1 | 8.9 | 0.12 | 0.73 |
